# Delayed Response After Confirmed Progression (DR) and Other Unique Immunotherapy-Related Treatment Concepts in Cutaneous Squamous Cell Carcinoma

**DOI:** 10.3389/fonc.2021.656611

**Published:** 2021-04-15

**Authors:** Annette M. Lim, Karda Cavanagh, Rodney J. Hicks, Luke McLean, Michelle S. Goh, Angela Webb, Danny Rischin

**Affiliations:** ^1^ Department of Medical Oncology, Peter MacCallum Cancer Centre, Melbourne, VIC, Australia; ^2^ Sir Peter MacCallum Department of Oncology, Faculty of Medicine, Dentistry and Health Sciences, The University of Melbourne, VIC, Australia; ^3^ Department of Cancer Imaging, Peter MacCallum Cancer Centre, Melbourne, VIC, Australia; ^4^ Department of Nuclear Medicine, Peter MacCallum Cancer Centre, Melbourne, VIC, Australia; ^5^ Department of Dermatology, Peter MacCallum Cancer Centre, Melbourne, VIC, Australia; ^6^ Department of Plastic Surgery, Peter MacCallum Cancer Centre, Melbourne, VIC, Australia

**Keywords:** immunotherapy, PD-1 inhibition, pseudoprogression, cutaneous squamous carcinoma, second primary tumors (SPTs)

## Abstract

Non-melanoma skin cancers are one of the most common cancers diagnosed worldwide, with the highest incidence in Australia and New Zealand. Systemic treatment of locally advanced and metastatic cutaneous squamous cell carcinomas has been revolutionized by immune checkpoint inhibition with PD-1 blockade. We highlight treatment issues distinct to the management of the disease including expansion of the traditional concept of pseudoprogression and describe delayed responses after immune-specific response criteria confirmed progressive disease with and without clinical deterioration. We term this phenomenon “delayed response after confirmed progression (DR)”. We also discuss the common development of second primary tumors, heterogeneous disease responses, and expanding clinical boundaries for immunotherapy use.

## Introduction

Non-melanoma skin cancers (NMSC) predominantly basal cell carcinomas (BCC) and cutaneous squamous cell carcinomas (CSCC) are the most frequently diagnosed cancer in North America and Australia/New Zealand (1). Although most are resectable, the morbidity related to disease is significant and accounts for the most common cancer-related cause for hospitalization in Australia exacerbated by the multiplicity of skin cancer excisions (2–4). Approximately 5% of CSCC recur or metastasize leading to death or management associated with significant morbidity due to disease occurrence on sun-exposed areas such as the face, head and neck (5, 6).

In 2018, the first report of the efficacy of cemiplimab, a PD-1 inhibitor, was published for the treatment of patients with locally advanced or metastatic CSCC who were not candidates for curative surgery or radiation. The objective response rate (ORR) to therapy was 47% (range: 34-61), leading to Food and Drug Administration (FDA) and European Medicines Agency (EMA) approval and a paradigm shift in the management of these tumors (7, 8). Updated data indicates that the median duration of response and median overall survival (OS) have not been reached, with estimated 24 month-OS being 73.3% (95% CI: 66.1-79.2) (9). The KEYNOTE-629 study showed that pembrolizumab is also efficacious (ORR 34%; 95% CI: 25- 44) (10), which also led to FDA approval. First line pembrolizumab in the CARSKIN trial for locally advanced or metastatic CSCC (including radiotherapy naive patients, n=20/57) achieved a week 15 ORR of 41% (95% CI: 26-58%) (11). Therefore, the use of PD-1 blockade for the treatment of advanced CSCC represents a major breakthrough in the management of these common epithelial cancers.

We report our immunotherapy management experiences unique to CSCC that challenge and expand current clinical concepts in practice.

## Expanding on the Concept of Pseudoprogression – “Delayed Responses After Confirmed Progression (DR)” and Response After Clinical Deterioration

The Response Evaluation Criteria in Solid Tumors (RECIST) criteria is a validated measure for the standardized evaluation of cancer therapies, determined by the assessment of the change of tumor burden with treatment (12). There are notable limitations of the RECIST guidelines in patients treated with immunotherapy, given that “pseudoprogression” can occur with an increase in tumor size due to inflammatory cell infiltrates followed by tumor reduction (13), and improved OS can occur without RECIST defined reduction in tumor measurements (14–16). Thus, RECIST criteria have been modified to include immune-related response criteria (irRC), immune-related response evaluation criteria in solid tumors (irRECIST), modified RECIST1.1 for immunotherapy (iRECIST), and immune-modified RECIST (imRECIST) (17–20).

Pseudoprogression occurs in under 10% of all cancers treated with immunotherapy, with an incidence in head and neck cancer of approximately 1% (21–24). To address pseudoprogression, the irRC, irRECIST and iRECIST require the use of confirmatory imaging at least 4 weeks after initial progression is documented (we will refer to this collectively as iCPD, immune confirmed disease progression as per iRECIST), a minimum size increase of >5x5mm^2^ or 10mm, an increase in the sum of tumor measurements from the nadir of 20-25%, and incorporation of new lesions in the sum of tumor dimension measurements before confirmation of progressive disease (PD) (17–19). The imRECIST criteria is similar, but permits the best response assessment to occur after observation of progressive disease, which avoids underestimation of survival rates (20). All criteria recommend that treatment beyond progression should only occur if a patient’s performance status and disease-related symptomatology are stable.

We describe two cases to redefine our understanding of pseudoprogression, with delayed disease response observed after iCPD and after observation of clinical deterioration. We introduce new terminology to capture the phenomenon as “delayed response after confirmed progression (DR)”. We also discuss a case of tumor response observed in a patient with clinical deterioration, with treatment beyond progression.

### Case One

This case illustrates the observation of DR. Patient One was diagnosed with a T2N0M0 occipital scalp/vertex CSCC excised with clear margins in September 2017. In July 2018, a 35mm in-transit recurrence was resected with an involved margin. Re-excision and ipsilateral neck dissection demonstrated a 2.5mm residual CSCC 1.2mm from the deep margin, and 1/41 nodes involved with extranodal extension to the surgical margin. Adjuvant radiotherapy of 66 Gy/33# was completed in October 2018. Within three weeks, biopsy of multiple new in-field in-transit CSCC and an out-of-field intramuscular lesion confirmed recurrent disease. Following multidisciplinary meeting (MDM) discussion, he was referred for consideration of immunotherapy for his recurrent CSCC.

**Figure 1:**
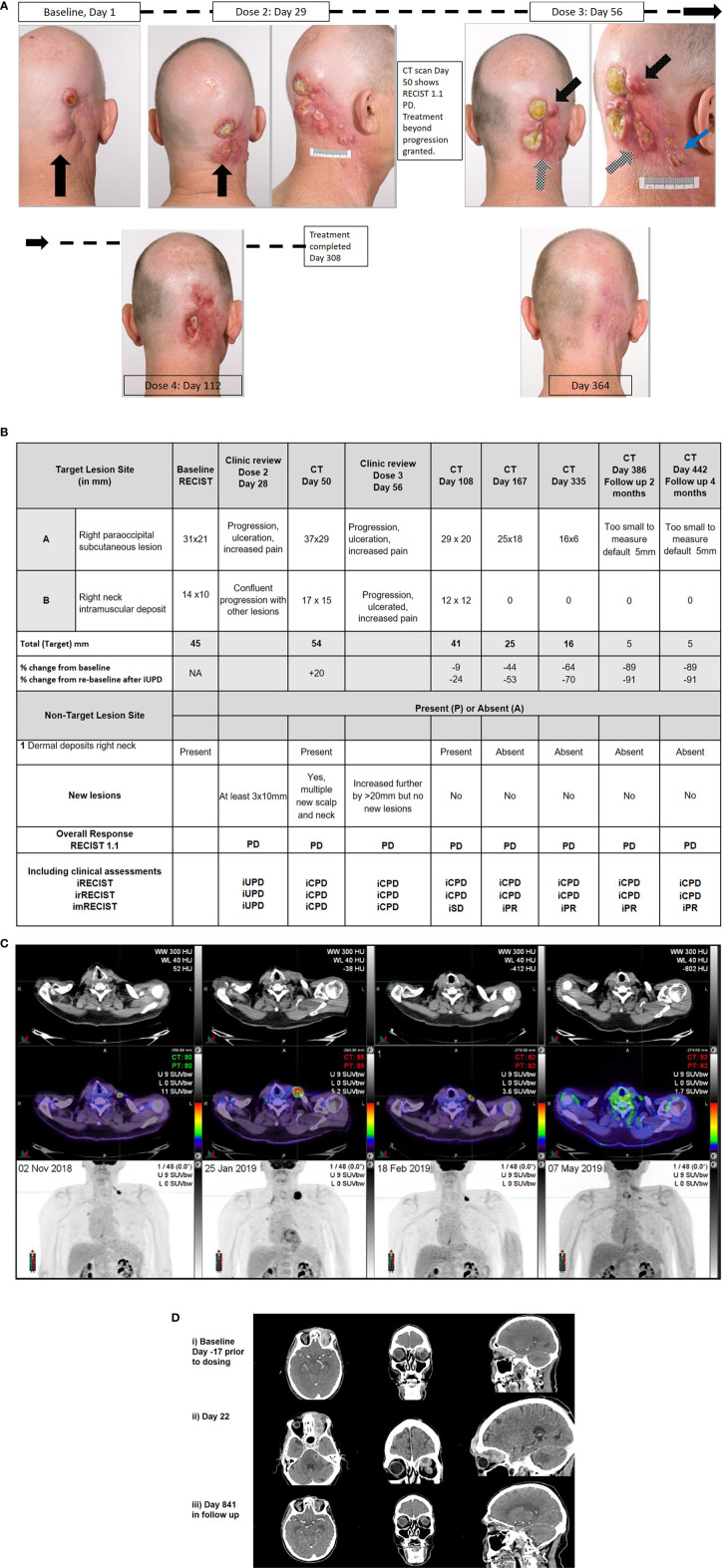
Correlative clinical photographs, tumor response measurements according to time, and fluorodeoxyglucose - positron emission tomography (FDG-PET) and CT scan images of discussed patient cases. **(A, B)** Patient One’s photographic images demonstrating iUPD and iCPD, with accompanying tumor response measurements according to RECIST 1.1 and immune based criteria at key time points. **(C)** Patient Two’s FDG-PET images over time with low dose axial CT, fused axial CT, and maximal intensity projection (MIP) images in each column from 02/NOV/2018, 25/JAN/2019, 18/FEB/2019, and 07/MAY/2019, demonstrating evidence of progression and regression with ongoing immunotherapy. **(D)** Patient Three’s representative images of disease at baseline, at progression and with response. Images i) Baseline (Day-17 prior to dosing) Post contrast CT: (axial, coronal, sagittal) demonstrates enhancing soft tissue extending from the cutaneous left forehead along the roof of the left orbit in the distribution of V1, involving the cavernous sinus, Meckel’s cave, nerve root entry zone of the left trigeminal nerve and likely involvement of the trigeminal nuclei with soft tissue at the left pons. ii) Day 22 Post contrast CT: (axial, coronal, sagittal) demonstrates increasing volume of enhancing soft tissue extending from the cutaneous left forehead along the roof of the left orbit in the distribution of V1, involving the cavernous sinus, Meckel’s cave, nerve root entry zone of the left trigeminal nerve and involvement of the trigeminal nuclei with soft tissue at the left pons. iii) Day 841 (in follow up) Post contrast CT: (axial, coronal, sagittal) demonstrates small volume residual non-enhancing soft tissue extending from the cutaneous left forehead along the roof of the left orbit. The remaining soft tissue has resolved on CT.

The patient was enrolled on the NCT02760498 to receive cemiplimab 400mg Q4W, with RECIST 1.1 measurements determined by radiological assessments as per protocol. The progression of lesions documented by photography, and radiological assessments are summarized in **Figures 1A, B**. Four weeks after initial dose, Patient One reported worsening disease-related pain. The largest CSCC lesion had increased by 10mm with new ulceration (black arrow), other baseline lesions had also increased in size with ulceration, while at least three new lesions greater than 10mm had developed with multiple other smaller lesions visible. This corresponded to unconfirmed progressive disease (iUPD). Four weeks later prior to his third dose, ongoing progression was noted with further ulceration and coalescence of lesions (white checked arrow), and increasing size of new nodules by more than 10mm (black spotted arrow) confirming iCPD. At that time point, RECIST 1.1 assessment by imaging confirming PD (**Figure 1B**). Approval for treatment beyond progression was granted. By the next visit, all lesions had improved clinically with a reduction in size and improvement in pain. The patient completed 12 months of therapy with a complete clinical response. To date, the patient remains in clinical and radiological remission.

### Case Two

This case illustrates DR. Patient Two had a long history of multiple NMSC lesions being excised from the head, neck, and chest, including a Merkel cell carcinoma (MCC). Patient Two had a CSCC lesion from the left clavicular area that required multiple re-excisions over a six-month period before clear margins were achieved in January 2018. In May 2018, a left infraclavicular chest recurrent CSCC was excised that involved the clavicular head of the pectoralis major muscle and pathologically measured 32mm with a deep margin of 0.7mm and evidence of perineural invasion. Re-excision demonstrated no residual disease but another 0.3mm central chest poorly differentiated carcinoma of uncertain origin was concurrently excised with clear margins. Adjuvant radiotherapy of 60Gy/30# was completed on August 2018. Two months later, a biopsy of a nodule over the medial aspect of the left clavicle confirmed recurrent CSCC. Magnetic resonance imaging (MRI) and computer tomography (CT) scans were unable to define the lesion, but fluorodeoxyglucose positron emission tomography (FDG-PET) scan identified an ~13mm left supraclavicular lesion (**Figure 1C**). After MDM discussion, Patient Two was referred for consideration of immunotherapy. Given his history of MCC, he was ineligible for trial participation and self-funded pembrolizumab 200mg Q3W which commenced on 28/NOV/2018. At review prior to the second dose (19/DEC/2018), his lesion had increased by more than 10mm to ~40x30mm with purpuric discoloration of the intact skin consistent with iUPD. By review prior to the third dose (08/JAN/2019), the lesion had fungated through the skin. A restaging FDG-PET scan (25/JAN/2019) performed after administration of three doses of pembrolizumab demonstrated marked interval progression with increase in size of disease to 33mm confirming iCPD (+254%). On 29/JAN/2019, iCPD was confirmed clinically and the fourth dose of immunotherapy was abandoned and surgical salvage was planned. Two weeks later prior to surgery, the patient reported clinical improvement and repeat imaging performed (18/FEB/2019) demonstrated reduction in tumor size to 15mm (-55% from previous). Pembrolizumab was resumed and by the subsequent visit, he had obtained a complete clinical response. Approximately 12 months of treatment was completed on 02/JAN/2020 and the patient remains in complete metabolic, radiological and clinical response to date.

### Case Three

This case highlights that clinical deterioration may not militate against disease response with the use of immunotherapy for the treatment of CSCC. Patient Three had a long history of multiple NMSC and at age 81 years was diagnosed with a T4N0M0 left supraorbital/intraorbital CSCC with perineural disease that involved all branches of the left trigeminal nerve to the cisternal portion. Despite 54Gy in 27 fractions of radiotherapy completed on December 2017, MRI scan in February 2018 revealed disease progression with increasing tumor anterior to the left frontal bone, new marrow infiltration of the floor of the left cranial fossa with nodular dural enhancement and no response in the pre-existing orbital disease (**Figure 1D**). After MDM discussion, he was referred for consideration of immunotherapy. He consented to trial participation in the NCT02760498 (cemiplimab 3 mg/kg Q2W). At Day 1 review, his ECOG performance status was assessed as 1, and examination demonstrated a 25mm left supraorbital mass, complete left eyelid proptosis, complete opthalmoplegia of his left eye, decreased sensation in the trigeminal nerve distribution with patient report of neuropathic pain. At Day 15 his pain had improved but he reported falls at home on the background of worsening balance, resulting in local trauma to the forehead. Clinical examination demonstrated no obvious abnormalities compared to baseline, however ECOG performance status was assessed as 2. Patient Three was referred for allied health management and received his second dose of cemiplimab. Two days later, he was admitted to hospital following a fall with a head strike with loss of consciousness, and reported nausea. CT brain (Day 21) identified disease progression (+44%, iUPD) with enhancement of the left trigeminal nerve disease into the cerebellar peduncle and left-sided pons with surrounding edema. The patient was discharged mobilizing on a wheeled frame the following day. At Day 29 review, the patient reported a significant improvement in his neuropathic pain, the supraorbital mass was clinically smaller but his ECOG performance status was assessed as 2. Restaging CT scans performed (Day 50) identified ongoing disease progression with an increase in size in all target lesions (+42% from baseline, RECIST 1.1 PD, iUPD). MRI brain demonstrated progression with increased diffuse skin thickening, increased enhancement of the orbital and periorbital disease, new destruction of the bones associated with the left frontal and ethmoid sinus and left orbit, soft tissue intracranial extension into the left anterior and middle cranial fossa, with increased trigeminal perineural invasion into the left brainstem. In clinic on Day 57, he reported a complete absence of trigeminal nerve pain and a reduction of the left supraorbital mass to 15mm was noted. Treatment beyond progression was approved. By Day 104, imaging demonstrated ongoing RECIST PD (+28%, iUPD) with subsequent review confirming regression of the exophytic component of the supraorbital CSCC and exposed bone. Patient Three went on to complete 659 days on therapy after experiencing a number of immune-related (≤Grade 2) and other medical adverse events (not treatment-related). His most recent imaging of his target lesions (**Figure 1D**) demonstrated complete resolution of the pons lesion, stable orbital lesion and “not measurable” ulcerated left scalp lesion.

Two of the patient cases presented introduce a new phenomenon we have defined as a “delayed response after confirmed progression (DR)”. That is, the observation of a clinical and radiological response after iCPD. In both cases, clinical deterioration was observed early in treatment but after treatment beyond progression, durable clinical and radiological improvement was obtained. In one case, change of tumor evolution with the cessation of new lesions developing may have been the only indication to herald DR. Review of our institutional experience of patients treated with cemiplimab on trial (up to 15/SEP/2020) in the advanced setting who have received more than one dose, demonstrates an estimated incidence of 2/39 (5%) of DR without any cases of “traditional pseudoprogression”. We have observed DR occurring late in treatment courses and “traditional pseudoprogression” for non-trial patients. We also raise the concept that clinical deterioration may not militate against tumor response. In distinction to the three cases discussed where all patients experienced disease-related deterioration, the KEYNOTE-629 study which used RECIST 1.1 criteria for response evaluation reported that 29 *clinically stable* patients received treatment beyond progression (10). Of these, 12 patients continue on therapy and eight patients have developed responses (1 complete response, 7 partial responses according to irRECIST; FIG S4 (10)).

Therefore, our observation of DR and scenarios discussed further below, caution against a nihilistic approach to CSCC patient management when using immunotherapy. These cases highlight the important consideration of the timing and method of disease response assessments and the limitations of most criteria to capture pseudoprogression/DR, response rates and best overall response (BOR) assessments as part of clinical trial reporting. Use of the RECIST 1.1 framework will have not accurately reflected BOR for these patients, with the known limitation of most frameworks to act as a surrogate for progression-free and overall survival (25). Although the timing of imaging assessments are necessarily at a time point to permit sufficient receipt of therapy to assess response, these scans may not contemporaneously reflect disease evolution and response. Further, data from trials that utilize immunotherapy in the neoadjuvant setting prior to definitive surgery suggests that complete pathological responses may be seen only as partial response on imaging (26–29). This highlights the clinical need for research into the predictive and prognostic role of imaging techniques in patients treated with immunotherapy for CSCC and the need to identify relevant molecular liquid biopsy biomarkers for disease surveillance (30–32).

## Second Primary Tumors on Immunotherapy

Multiplicity is common for patients with NMSC, with ~74% of all skin cancers being excised from patients with multiple NMSC lesions particularly involving the head and neck region (2, 3). We have observed that patients on immunotherapy can develop both CSCC and BCC as second primary tumors (SPT) despite responsive disease elsewhere, likely due to field cancerization (33). The mechanism by which SPT escape treatment control have not yet been elucidated and are likely to illuminate molecular mechanisms behind immune escape.

From a clinical management perspective, it is critical that SPT development is not mistaken as treatment failure given that these lesions can resolve with ongoing therapy or can be managed with local therapy. As a general principle, the SPT should be observed for a period whilst continuing immunotherapy and if regression or stability does not occur then local therapy can be pursued with a view of continuing systemic therapy for control of the other immunotherapy-responsive disease. On retrospective review of our patients treated with cemiplimab on trial, of eight patients who developed biopsy proven SPT resistant to immunotherapy, seven patients with CSCC and six with BCC required formal excision and/or radiotherapy for local management with all but one patient having more than one lesion. We have not identified any situation for which the new SPT has resulted in metastatic disease or has heralded the development of disease progression in existing lesions. Molecular profiling of these SPTs that develop on immunotherapy is important to define disease heterogeneity and to identify the likely numerous mechanisms of immune escape.

## Heterogeneous Responses on Immunotherapy

Discordant immunotherapy responses can be observed between existing lesions, where concurrent local therapy for immunotherapy-resistant lesions may be warranted to secure control. Given the common multiplicity of NMSCs occurring in the same patient (2), awareness of response heterogeneity is key to avoid inappropriate early cessation of immunotherapy.

### Case Four

This case demonstrates heterogeneous responses of two baseline lesions to immunotherapy. Patient Four was a 70 year old man with a multiply recurrent CSCC of his left forearm which had required six re-excisions. The largest resected recurrence was a 70x50mm spindled/poorly differentiated CSCC, up to 8mm in depth, with lymphovascular and perineural invasion. Adjuvant 56Gy/28# of radiotherapy was completed in September 2018, with a truncated course due to toxicity. In May 2019, Patient Four developed a painful locally recurrent CSCC of his left elbow with bone on view that measured 41x32mm on MRI scan, and a small right cheek lesion suspected to be a separate primary CSCC. Following MDM discussion, surgery for the cheek lesion was planned prior to immunotherapy for the elbow lesion. Wide local excision of the cheek lesion on the 26/JUN/2019 revealed three areas containing moderately differentiated CSCC spanning over 26mm, 32mm and 40mm with a transected deep margin, multiple CSCC tissue deposits of 2mm to 8mm, with perineural invasion abutting the margin. Re-excision was performed on 03/JUL/2019 revealing multiple foci of moderately differentiated CSCC deposits with perineural invasion, vascular tumor emboli, and tumor 0.2mm from a margin. Patient Four consented to participation the NCT02760498. At baseline, the exophytic left elbow lesion clinically measured 70x35mm. Prior to the third dose (Day 57), a near complete clinical response of the left elbow CSCC was observed with a residual superficial ulcer measuring 10mm accompanied by an improvement in pain. MRI scan confirmed the lesion had reduced to 32x13mm. A month later (Day 87) a right cheek nodule recurred and given its persistence, resection was performed on Day 106 demonstrating an 11mm CSCC with ≤1mm margins. Ongoing immunotherapy secured a complete clinical response for the left elbow lesion by Day 141 with imaging showing no identifiable tumor (nominal RECIST measurement of 5mm). However, three further excisions (Day 147, Day 188, Day 218) were required to manage the cheek lesion which recurred twice during adjuvant radiotherapy planning. Palliative radiotherapy of 36Gy in 6# to the cheek lesion was completed on Day 283. Most recent imaging demonstrated a complete metabolic response (Day 335) and ongoing radiological PR (nominal 5mm, Day 447) of the left elbow disease. However, since Day 394 clinical recurrence of the right cheek nodule has been progressive. Due to the absence of local therapies available to effectively treat the recurrent right cheek CSCC, Patient Four decided to cease further immunotherapy to pursue best supportive care.

Tumoral heterogeneity leading to discordant treatment response is a known therapeutic hurdle that can contribute to disease progression with the development of clonal resistance (34–38). In the era of immunotherapy which can secure durable disease control, understanding the contribution of local therapy towards overall disease control and survival is crucial but poorly understood. It has been observed in patients with melanoma treated with immunotherapy who received local therapy to progressive lesions in order to achieve no evidence of disease, that those who had local therapy to new lesions had poorer survival compared to those who had local therapy to progressive pre-existing metastases (PFS 6% vs 70%, p=0.001) (39). Consideration of the anatomical site of oligoclonal resistant disease will have clinical (e.g. brain versus lung) and therapeutic implications (e.g. surgery versus radiotherapy). Patient Four’s case demonstrates that discordant immunotherapy responses can be observed between lesions, where concurrent local therapy may be warranted in an attempt to secure control of immunotherapy-resistant lesions.

## Expanding Clinical Boundaries - Activity in Advanced, Fungating, Disfiguring Disease and Good Tolerance Despite Multiple Comorbidities

It is essential to consider patient factors, treatment morbidity and goals of management in oncological care. Comorbidities and ECOG performance status guide management to optimize patient outcomes that may focus on quality of life or may be driven to obtain survival benefits or both. It is well recognized that a large disparity exists between trial patients versus “real-world” patients, who often are older, of minority groups, and with comorbidities that may interfere with assessment of therapeutic efficacy or toxicity (40) (41, 42). In the context of the functionally and cosmetically sensitive anatomical region of the head and neck, it is crucial to define that immunotherapy is generally tolerable and associated with improved quality of life (22, 43, 44). In CSCC, Maubec et al. have reported improved health-related quality of life for patients with immunotherapy-responsive disease (11), and use of PD-1 blockade has been demonstrated to be tolerable with side effects reported similar to other checkpoint inhibitors and with the ability to secure durable disease control (7, 9, 45). Noteworthy in the trial reports, is the median age of patients being 71-80 years with the oldest patients being 99 years old (7, 10, 11).

**Figure 2:**
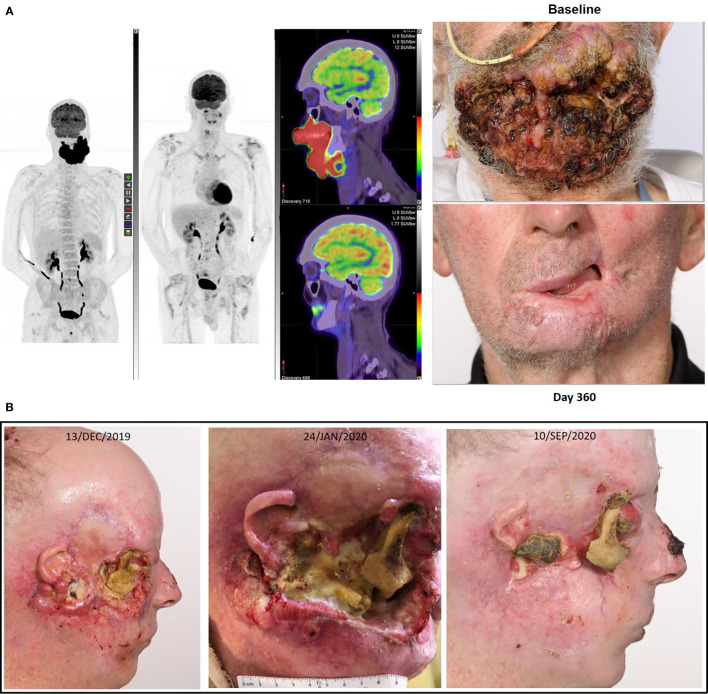
**(A)** This figure illustrates the FDG-PET matched MIP and fused axial images with photographs at baseline and after receipt of cemiplimab of a 75-year-old male who had declined investigation and treatment of CSCC originating from his chin. After 18 months of pursuing alternative treatment, he accepted immunotherapy when the disease had become so advanced it mechanically impacted his ability to eat. He consented to participation in the NCT02760498, and after receipt of two doses clinical regression of the lesion was noted. His disease remains in complete remission after two years of therapy completed more than 12 months ago. **(B)** This figure illustrates re-epithelialization occurring during the receipt of compassionate access cemiplimab in a 45-year-old patient with more than a 20 year history of multiple NMSC including synchronous CSCC, BCC and MCC. The patient ceased vismodegib to commence cemiplimab on 13/DEC/2019 but required recommencement of vismodegib on 19/APR/2020 due to recurrence of multiple BCC lesions. He remains on dual therapy given the symptomatic improvement achieved with good pain control and resolution of right cheek CSCC-related trismus.

Our institutional experience in the trial and “real-world” setting is that checkpoint inhibitor therapy is exceptionally well tolerated by patients with CSCC. Generally, few treatment contraindications exist and few comorbidities raise concern including extreme age, dialysis, other synchronous malignancies requiring treatment, and poor ECOG performance status. This is a paradigm change in our approach to patients with CSCC and is paramount given the dramatic response rates achieved by therapy, providing symptomatic and durable control. This is in stark contradistinction to our approach with mucosal head and neck cancer patients. As illustrated in **Figure 2**, there are not many clinical situations in which disease is considered “too advanced” for immunotherapy. That is, the stage of disease, location of disease, and extent of disease does not militate against response to immunotherapy. Anecdotally, the extent of re-epithelialization following response can be impressive creating complexity around the timing of reconstructive surgery if pursued.

## Conclusion and Future Directions

Use of immunotherapy has revolutionized the care of patients with advanced CSCC, leading to a paradigm shift in the selection of patients for treatment with the expectation of response and durable control even in the advanced recurrent or metastatic setting. We have focused on describing unique clinical concepts related to the treatment of CSCC with immunotherapy including the phenomenon of delayed response after confirmed progression (DR), observation of tumor responses despite clinical deterioration in iCPD, and the need to consider flexible treatment approaches in patients with multiple NMSC.

An improved understanding of CSCC will undoubtedly enhance patient selection for therapy as ongoing clinical research efforts investigate the role of immunotherapy in the adjuvant and neoadjuvant settings. Few cancer registries collect data on CSCC or advanced CSCC, limiting our understanding of the spectrum of disease, burden of need, morbidity and costs related to treatment. The therapeutic advances necessitate rapid development of real-time methods to assess tumor response (e.g. liquid biopsy, imaging or combination approaches) that are more informative than current imaging modalities and response criteria. Translational research will be crucial to molecularly define the clinical spectrum of CSCC (46, 47), and identify reliable predictive and prognostic markers to therapy, including mechanisms of immune evasion. Specifically, comprehensive profiling of immunotherapy exposed tumors, including single cell sequencing approaches, will be important to further clarify inter-tumoral, intra-tumoral and tumor microenvironment molecular processes that underpin the described clinical concepts (48, 49).

## Data Availability Statement

The original contributions presented in the study are included in the article. Further inquiries can be directed to the corresponding author.

## Ethics Statement

The studies involving human participants were reviewed and approved by Peter MacCallum Cancer Centre Ethics Committee. Written consent has been provided by the patient with reidentifiable images.

## Author Contributions

Concept and design – AL, DR. All authors contributed to the article and approved the submitted version.

## Funding

DR is supported by a NHMRC Investigator Grant APP1175929.

## Conflict of Interest

AL –Uncompensated advisory board7 from Merck Sharp & Dohme and Bristol-Myers Squibb with travel and accommodation expenses; uncompensated consultancy for Eisai. RH - Shareholder in Telix Pharmaceuticals with honoraria and any dividends donated to the Peter MacCallum Cancer Centre. DR - Institutional research grant and trial funding from Regeneron Pharmaceuticals, Inc., Roche, Merck Sharp & Dohme, Bristol-Myers Squibb, and GlaxoSmithKline, Sanofi; uncompensated scientific committee and advisory board from Merck Sharp & Dohme, Regeneron Pharmaceuticals, Inc., Sanofi, GlaxoSmithKline, and Bristol-Myers Squibb, and travel and accommodation from Merck Sharp & Dohme and GlaxoSmithKline.

The remaining authors declare that the research was conducted in the absence of any commercial or financial relationships that could be construed as a potential conflict of interest.
